# An Emergency Switch to Distance Learning in Response to the COVID-19 Pandemic: Experience from an Internationally Accredited Undergraduate Pharmacy Program at Qatar University

**DOI:** 10.1007/s40670-020-01079-9

**Published:** 2020-09-11

**Authors:** Farhat Naz Hussain, Reem Al-Mannai, Abdelali Agouni

**Affiliations:** College of Pharmacy, QU Health, Qatar University, P.O. Box 2713, Doha, Qatar

**Keywords:** COVID-19, Distance learning, Educational emergency, Pandemic, Technology-enhanced learning

## Abstract

The world is experiencing an unprecedented public health emergency owing to the COVID-19 pandemic. To control virus spread, many countries temporarily suspended classes. In this context, the availability of e-tools and distance learning platforms in higher education institutions has proven very useful to facilitate the emergency switch to distance learning to ensure continuity of the educational process. We discuss here the experience of the College of Pharmacy at Qatar University in responding to suspension of classes using available educational technologies. Furthermore, we provide some reflection points for optimal implementation of technology-enhanced learning into distance education for future academic years.

## Introduction

In the past decade, the use of various technologies to support the process of teaching and learning has rapidly developed in higher education, with several technologies and e-tools being currently employed to facilitate teaching and promote active learning in classroom settings [1]. The use of technology to enhance the learning experience for students is becoming an integral part of the teaching strategies followed by instructors worldwide, encouraging collaboration, active learning and a student-centred learning experience [2]. Besides supporting students’ learning inside and outside of classroom settings and facilitating the delivery of various innovative teaching methods such as flipped classrooms [2], the availability of a variety of technological tools, such as live teaching platforms and lecture capture systems, also supported the use of distance learning within higher education. Many institutions worldwide are using online or a blended learning approach especially with the fast growth of massive open online courses (MOOCs) [3, 4]. Motivations for offering programs via the distance learning approach vary dramatically and include financial and logistical reasons such as constrained classroom availability and to facilitate access to programs to full-time employed students. The number of students enrolled in distance learning higher education programs is increasing and, in some programs, outpaces the traditional teaching enrollment [5].

In the past months, the world has experienced an unprecedented public health emergency owing to the new coronavirus disease (COVID-19) pandemic that started in China and rapidly spread to most countries across the globe [6–8]. Many countries around the world have decreed unprecedented preventive measures to control the spread of the virus among which include the temporary closure of schools and universities in specific regions/cities or nationwide [9]. In the context of this pandemic, the availability of e-tools and distance learning platforms in higher education institutions has proven very useful to facilitate the emergency switch to distance learning. The prior availability and exposure of faculty and students to these e-tools may have facilitated the emergency switch to distance learning and ensured the continuity and quality of the educational process. In this article, we reflect on the experience of the College of Pharmacy at Qatar University in responding to the suspension of classes using available educational technologies and how the past experience of the College in using technology-enhanced learning (TEL) had a positive impact on this response and moderated the adverse effects on students’ learning experience. We provide some reflection points for optimal implementation of TEL into distance education for future academic years.

The State of Qatar, in a precautionary measure to control the spread of the virus, suspended classes in all schools and universities nationwide from March 9, 2020, until further notice [10]. To continue providing a high-quality learning experience to all pupils, several steps were taken by all private and public schools to continue educational activities using online tools and virtual learning environment (VLE) platforms.

With a specific focus on the higher education sector, Qatar University, as the main national University, has taken immediate action to optimize a variety of e-tools currently available for collaboration and communication, including Blackboard Collaborate Ultra (BCU) (an interactive online lecture delivery system) that is embedded within the University VLE system (Blackboard 9.1), and conferencing tools such as WebEx, Zoom and Microsoft Teams. These tools allow faculty, staff members and students to host or join online meetings, access virtual study rooms with audio and high definition (HD) video and allow application sharing and session recording capabilities from any computer, smartphone or tablet devices. Immediately after the government’s decision to suspend classes, additional workshops and training sessions on the optimal use of these technologies were organized to resume teaching/learning activities and minimize impact on students. Faculty members immediately engaged in this institutional effort and multiple group discussions were organized to identify the best technologies to be used and best formats to be followed for each type of educational activity and style (lecture, team- or problem-based learning, group work, presentations, clinical skills, laboratory practical sessions...etc). A focal person for each college was appointed to answer any technical queries faculty may have on the use of the online systems. Faculty members at the College commented on the positive switch to distance learning:

“With the advent of COVID-19, there was a need for an instant switch to distant learning, and suddenly instructors were at the mercy of technology to mitigate this urgent need. Fortunately, College of Pharmacy, Qatar University is equipped with the state-of-the-art modalities for remote delivery of courses effectively, boosted by training courses provided by the Center for Excellence in Teaching and Learning (CETL). Therefore, the transition into online delivery was seamless at the College. Personally, I found the online tools very rewarding in the delivery of professional practical classes (Pharmaceutics), where transferable skills traditionally rely heavily on in-person delivery. COVID-19 may remain with us in the foreseeable future and it is pleasing to note that the College of Pharmacy has put in place all necessary online modalities to ensure that the quality of teaching and student experience is not impacted significantly”.

“The COVID-19 pandemic hits mid-spring semester and teaching, learning and assessment as we knew it changed. The unforeseen disruptions were initially challenging but I was focused on the brighter side and took that rare opportunity to expand my capacity and develop new pedagogical skills in mastering the use of the varieties of digital platforms available for the virtual delivery of teaching, learning activities, and assessment of students’ progress with their learning. Overall, the digital switch was challenging but enriching and de-mystifying”.

## Reflection on the Emergency Switch to Distance Learning at the College of Pharmacy at Qatar University

The College of Pharmacy at Qatar University, where the Bachelor of Science in Pharmacy program is accredited by the Canadian Council for Accreditation of Pharmacy Programs (CCAPP) since its inception in 2008, has always been at the forefront of using technology to enhance the learning experience of undergraduate students. Several educational technologies are already systematically used across the program to assist with teaching and introduce active learning in classroom settings, but one of particular note which was proven to be very useful during the suspension of classes as a response to COVID-19 was the Echo360 lecture capture system. Audio and video lecture capture is recorded through the Echo360 system with an overlay of the presented PowerPoint presentation slides or whiteboard for all regularly scheduled classes. A file is created and posted online 24 hours after the session is complete through the VLE (Blackboard 9.1). Students have access to the archived lectures from previous academic years, until graduation. There are no limits placed on the number of times a student can view the capture and students may view all or a selected portion of the link. This system has been systematically used to record sessions for most undergraduate courses at the College of Pharmacy for the past 10 years and all lectures are archived and can easily be viewed by all users through the course page on the VLE [11].

As an immediate response to the suspension of classes and to avoid any interruption in the learning process for students, students were immediately directed to archived lecture captures for the affected sessions where students could review the content from past cycles. To complement the students’ experience, additional updated materials were posted on the course pages on the VLE and discussion fora for each session were created for direct interaction between students and instructors. The availability of archived lecture captures for the majority of courses across the program minimized the impact of class suspension on students especially that students are already familiar with the system, which is systematically implemented, and all students are trained to use. Students commented on the transition to distance learning and their experience:

“The transition was challenging and hard at first but luckily the College of Pharmacy at Qatar University were already using Echo360 and recorded lectures before the pandemic. This archive of lectures became very useful during the transition as we students used it and were already familiar with the archive, until us and faculty members could adopt the new live online teaching”.

“Using recorded lectures and archived lectures did help in making sure that I was able to learn during this unexpected situation. Such sessions were able to deliver the information successfully but lacked student interaction and the opportunity to ask questions. However, it did help in providing a smooth transition, especially until starting real-time sessions”.

“While Blackboard collaborate ultra sessions were not the same as being in class, I think it was the best medium to deliver online sessions. The pros were that I was able to attend classes from home which saves time, still have interaction and ask questions using the microphone or chat tools, and listen to the sessions again if needed, since they were recorded. However, there were technical difficulties with Blackboard crashing which was an inconvenience. Furthermore, it was not possible to create the same environment as the classroom which made it more challenging to learn. Lastly, it was more difficult to understand lectures in which there were calculations such as therapeutic drug monitoring”.

“I feel that I did not learn much when it came to labs. Since they were online, I did not get the chance to practice any experiments or have counselling sessions. I was still able to gain some skills such as writing standard operating procedure notes and reflections but hardly any practical skills”.

From our experience, we strongly recommend higher education institutions to implement processes to allow for lecture recording and archiving. On reflection, the availability of these lecture recordings significantly reduced the negative impact the suspension of classes may have had on students’ learning, while allowing the College to provide a rapid response to missed lectures and gain invaluable time to avoid issues of using live lecturing tools.

In parallel to using archived lecture captures and discussion fora, other technologies were used to facilitate remote delivery of lectures and other types of educational activities. For instance, lectures were delivered “live” using the BCU tool, which is embedded within the university VLE system. This system allows live interaction with students who can present, chat, ask and answer questions during the lecture session. The instructor and students also have access to a whiteboard and can share material such as lecture notes, videos or files. The session content can be recorded and become accessible to students to review off-line whenever they wish. On reflection, we note some points which should be taken into consideration for future online lectures. It is not possible to determine how a student is responding to the material taught online without the ability to see “live” facial expressions. Although students have the opportunity to ask questions throughout the lecture, the collaborative learning environment which occurs from students asking and answering questions is missing. Furthermore, faculty members must reconsider the time required to deliver online lectures compared with face-to-face delivery. More time may be required to answer questions in a systematic manner, whether through the chat function or by using the microphone. There may also be a lag time between faculty members changing slides and the students viewing them depending on the quality of the internet connection, resulting in an accumulation of extra time.

### Delivery of Practical and Group Sessions

For practical and group sessions, several strategies were followed to enhance the learning experience and reduce the impact of remote delivery. In clinical skills sessions, BCU was used where students were individually provided an opportunity to discuss with simulated patients and collect the required information (e.g., medication history for interactions…etc). These sessions unlike traditional face-to-face teaching required additional support from teaching assistants to facilitate and organize video-capture and student/instructor/simulated patient interactions. The switch to distance-based learning for patient assessment labs also resulted in course instructors virtually demonstrating patient assessment techniques using mannequins and pre-recorded videos. In pharmaceutical laboratory sessions, a video recording of the instructor conducting the experiment was posted online through the VLE and then students were provided with the experimental results. An assignment was set, and tutorial sessions were organized to ensure all students understood the concepts.

In a journal club setting, the BCU platform allowed students to connect and listen while the instructor moderated the session. While this is not optimal for journal club delivery, the “chat” and “raise hand” functions on the BCU system allowed students to participate and resulted in a smooth delivery of the session. When students are expected to present and moderate a journal club session, the BCU system allowed the instructor to change the role of the presenting student from “participant” to either “presenter” or “moderator”, which allows the student to handle the discussion with peers independently.

In sessions involving group discussion and small group collaboration such as integrated case-based learning, the available platforms, such as BCU or WebEx, allow instructors to breakout the class into smaller groups that are independent from the main room and assign participants to specific groups. These breakout groups have their dedicated audio, video, whiteboard, content sharing and chat tools. The activities conducted in the breakout groups are totally independent from each other and from the main room. The main room is used by instructors for general briefing and discussion involving the whole class.

### Assessments in the Context of Distance Learning

The assessment strategy at the College of Pharmacy was revised in light of the changes required for distance-based learning. Assessments were conducted throughout the spring semester using a variety of online tools such as Blackboard 9.1 (VLE) and Turnitin for assignments. Turnitin module embedded to the VLE system of the University was especially useful in providing student feedback and plagiarism checks which was of utmost importance during this time. Students had an opportunity to upload their course work assignments through Turnitin and obtain immediately a similarity report, an unlimited number of times up until the deadline. Each time they submit a new document, it writes off the previous one and instantly provides a new similarity report to the student, which helped the students to improve their writing as they progressed with the write-up rather than wait until the deadline and obtain a report to be used for summative evaluation. The intention was to use the Turnitin tool as a developmental and formative tool to help the students improve the quality of their academic write-up.

During the switch to distance learning following COVID-19 pandemic, all exams were conducted using the “assessments” tool embedded to Blackboard 9.1 (VLE). At the College of Pharmacy, for nearly a decade, all written exams were conducted online using this tool which allows the use of a variety of assessment methods, including multiple choice questions (MCQ), multiple answer questions, fill in the blank, jumbled sentence, matching, calculated formula, ordering, short answer questions and essays. This past experience with online exams meant that both students and faculty were already familiar with online examination process and tools. However, the major challenge was to conduct online examination with students outside of the campus and difficulties to invigilate the exam and ensure its integrity and also to address any individual requests from students during the exam time.

The majority of examinations were changed to online open book assessments and each exam underwent peer-review by a committee of experts for quality control, to assess the level of difficulty, to ensure alignment with course learning objectives and to assess appropriateness of time. While the students were attempting the exam through the VLE (Blackboard 9.1), they were also asked to connect through either Microsoft teams or WebEx to ensure effective communication with instructors and invigilators during the exam to receive exam instructors, ask questions or report any difficulties or technical issues they may face. Students were asked to switch on their cameras and to not use the microphone to avoid disturbing the group, and when they needed to ask questions, they could use the raise hand function or post their query through the chat box either to all the group or only to a specific instructor. Some points must be taken into consideration for future online examinations such as how to implement effective invigilation during examinations to avoid cheating and to ensure students submit their examination at the correct stated time. In this context, there are several tools available for remote proctoring and online invigilation such as ExamMonitor offered by ExamSoft, which allows continuous audio and video monitoring of students throughout the exam. This digital proctoring system has been successfully used for examining medical students at Qatar University.

### Challenges

The use of these distance learning platforms was not without issues. Connectivity issues relating to the high usage of the systems, when exceeding their capacity limits, and internet/Wi-Fi issues resulted in some students losing connection or unable to log in. To counter this issue, all sessions delivered online are recorded and posted through the VLE. Trial and error in the use of the system allowed faculty members to share tips and ideas for ease of use of the systems (i.e., all students should turn their microphone off unless speaking to ensure good sound quality in the session). It was difficult to provide the optimal learning experience with the practical laboratory experiments; however, faculty at the College aimed to overcome these barriers by using online videos and detailed handouts and offering additional support sessions and increasing office hours for students to ask questions. As the COVID-19 pandemic continues, we postulate more issues may arise, and hope to overcome these barriers and provide fruitful discussions and learning for students.

## Conclusion

Overall, this unique educational experience in an international health emergency context demonstrates the importance of TEL, not only to enrich the student’s learning experience but also to strengthen the capacity of higher education institutions to respond to educational emergencies in situations where physical presence of students is not possible or severely limited. The availability of these technologies for many years and their wide use across Qatar University in general and at the College of Pharmacy in particular has facilitated the quick transition to a high-quality distance learning mode despite our institution being a conventional one. This experience provides strong evidence of the importance of preparedness of higher education institutions to ensure continuity of teaching and learning activities by adopting educational technologies and embedding them into the curriculum side to side with traditional delivery methods. It appears critical now that traditional campus-based universities should consider delivering certain components of their programs in a distance learning format because of the multiple benefits it offers due to its flexibility, but also to ensure both faculty and students are trained and familiar with distance learning concepts, tools and challenges which will leverage the preparedness of the educational institution in case future emergency events occur and prevent face-to-face education.
